# Effects of Zinc Chelators on Aflatoxin Production in *Aspergillus parasiticus*

**DOI:** 10.3390/toxins8060171

**Published:** 2016-06-02

**Authors:** Josephine Wee, Devin M. Day, John E. Linz

**Affiliations:** 1 Department of Food Science and Human Nutrition, Michigan State University, East Lansing, MI 48824, USA; jmw544@cornell.edu (J.W.); murph218@msu.edu (D.M.D.); 2 Department of Microbiology and Molecular Genetics, Michigan State University, East Lansing, MI 48824, USA; 3 Center for Integrative Toxicology, Michigan State University, East Lansing, MI 48824, USA

**Keywords:** *Aspergillus parasiticus*, aflatoxin biosynthesis, zinc, zinc chelators

## Abstract

Zinc concentrations strongly influence aflatoxin accumulation in laboratory media and in food and feed crops. The presence of zinc stimulates aflatoxin production, and the absence of zinc impedes toxin production. Initial studies that suggested a link between zinc and aflatoxin biosynthesis were presented in the 1970s. In the present study, we utilized two zinc chelators, *N,N,N′,N′*-tetrakis (2-pyridylmethyl) ethane-1,2-diamine (TPEN) and 2,3-dimercapto-1-propanesulfonic acid (DMPS) to explore the effect of zinc limitation on aflatoxin synthesis in *Aspergillus parasiticus.* TPEN but not DMPS decreased aflatoxin biosynthesis up to six-fold depending on whether *A. parasiticus* was grown on rich or minimal medium. Although we observed significant inhibition of aflatoxin production by TPEN, no detectable changes were observed in expression levels of the aflatoxin pathway gene *ver-1* and the zinc binuclear cluster transcription factor, AflR. Treatment of growing *A. parasiticus* solid culture with a fluorescent zinc probe demonstrated an increase in intracellular zinc levels assessed by increases in fluorescent intensity of cultures treated with TPEN compared to controls. These data suggest that TPEN binds to cytoplasmic zinc therefore limiting fungal access to zinc. To investigate the efficacy of TPEN on food and feed crops, we found that TPEN effectively decreases aflatoxin accumulation on peanut medium but not in a sunflower seeds-derived medium. From an application perspective, these data provide the basis for biological differences that exist in the efficacy of different zinc chelators in various food and feed crops frequently contaminated by aflatoxin.

## 1. Introduction

Five billion people globally are affected by aflatoxin contamination in many important food staples including peanuts, corn, sunflower seeds, and tree nuts. Contamination with this fungal metabolite is not just a financial burden, but it has been reported to be carcinogenic to humans and livestock [1,2]. *Aspergillus parasiticus* and *A. flavus* are two fungal species predominately responsible for producing aflatoxin via an innate biosynthetic pathway. Activation of this pathway and subsequent production of aflatoxin has been linked to many external and internal influences including light exposure, oxidative growth conditions, fungal volatiles, and nutrient availability including sugars and metal ions such as zinc [3,4,5].

The metal ion zinc (Zn^2+^) is required for the production of aflatoxin [6,7,8,9]. Between the 1960s and 1980s, several studies described a stimulatory effect of zinc on aflatoxin biosynthesis in *A. parasiticus* and *A. flavus* [7,10,11,12]. Zinc is a ubiquitous and tightly regulated metal ion necessary for optimal fungal growth. The estimated concentration of zinc in cells is within the picomolar to nanomolar range [13]. Prior studies demonstrated that zinc depletion reduces or completely inhibits fungal growth [14]. The cellular zinc composition is closely mediated within the fungal cell by zinc-binding proteins and membrane transporters known as Zip transporters [13]. In a study conducted with *Cryptococcus gattii*, *N*,*N*,*N'*,*N′*-tetrakis (2-pyridylmethyl) ethane-1,2-diamine (TPEN) was utilized to create zinc deprivation and this resulted in the induced expression of genes, ZIP1, ZIP2, and ZIP3 encoding zinc transporters [15].

Aflatoxin biosynthesis is catalyzed by 25 or more enzymes encoded by up to 30 genes clustered in the sub-telomeric region of chromosome 3 in *A. parasiticus* and *A. flavus* [16,17]. Within aflatoxigenic aspergilli, the expression of a number aflatoxin biosynthetic pathway genes is positively regulated by the zinc binuclear Zn(II)2Cys6 cluster transcriptional regulator AflR further supporting the role of zinc in aflatoxin production at the molecular level [18].

Our laboratory has focused extensive research effort on identifying and characterizing compounds that inhibit aflatoxin biosynthesis [4,19,20,21,22,23,24]. Motivated by the stimulatory effects of zinc on toxin production, here, we utilized the zinc chelators, *N,N,N′,N′*-tetrakis(2-pyridylmethyl)ethane-1,2-diamine (TPEN) and 2,3-dimercapto-1-propanesulfonic acid (DMPS) to limit zinc availability during growth of *A. parasiticus* on laboratory media with the goal of inhibiting toxin production. Although zinc has been known to stimulate aflatoxin biosynthesis, we are the first to report the use of zinc chelators to deplete access to zinc and downregulate alatoxin synthesis on growth media and natural substrates.

In the current study, we found that TPEN but not DMPS significantly decreases aflatoxin production of *A. parasiticus* grown on laboratory media. Thus, we focused our efforts on characterizing the inhibitory effect of TPEN. TPEN treatments significantly decreased toxin production between 3 and 6 fold in *A. parasiticus* on rich and defined growth media. We hypothesized that zinc limitation would down-regulate function of zinc-dependent transcription factors, such as AflR, one key pathway regulator responsible for aflatoxin gene activation. Interestingly, we did not detect significant differences in transcript accumulation of several aflatoxin pathway genes including *aflR* in the presence of TPEN under the conditions tested. In order to investigate the effect of TPEN independent of gene expression, we used a fluorescent zinc probe to track intracellular levels of zinc. Preliminary observations suggest an increase in fluorescence intensity of the zinc probe, Zinpyr-1 added to fungal cells treated with TPEN. Lastly, we investigated the effect of TPEN on peanut- and sunflower seed-derived medium. We found that TPEN significantly decreased aflatoxin accumulation of *A. parasiticus* grown on peanuts but not on sunflower seeds. These differential effects of TPEN suggest important considerations for practical application of zinc chelators or other food-safe inhibitors that aim to block toxin production in the field and in storage.

## 2. Results

### 2.1. Effect of Zinc Chelators, TPEN and DMPS on Aflatoxin Accumulation and Growth of *A. parasiticus*

*A. parasiticus* wild-type strain, SU-1 was grown for 50 h with the standard growth protocol on solid PDA media containing varying concentrations of TPEN (20 μM, 100 μM, 500 μM), 50 μL of DMSO (vehicle control) or no addition (negative control). Preliminary dose response experiments demonstrated that 500 μM TPEN completely inhibited fungal growth on solid PDA medium (Figure S1). 100 μM TPEN significantly decreased aflatoxin production without affecting fungal growth as measured by fungal colony diameter (see Methods and Figure S2, Panel A), whereas 20 μM TPEN treatment did not affect aflatoxin accumulation on solid PDA medium as measured by TLC analysis (Figure S2, Panel B). Therefore, 100 μM TPEN treatment was used as the effective inhibitory dose for subsequent experiments on PDA.

**Figure 1:**
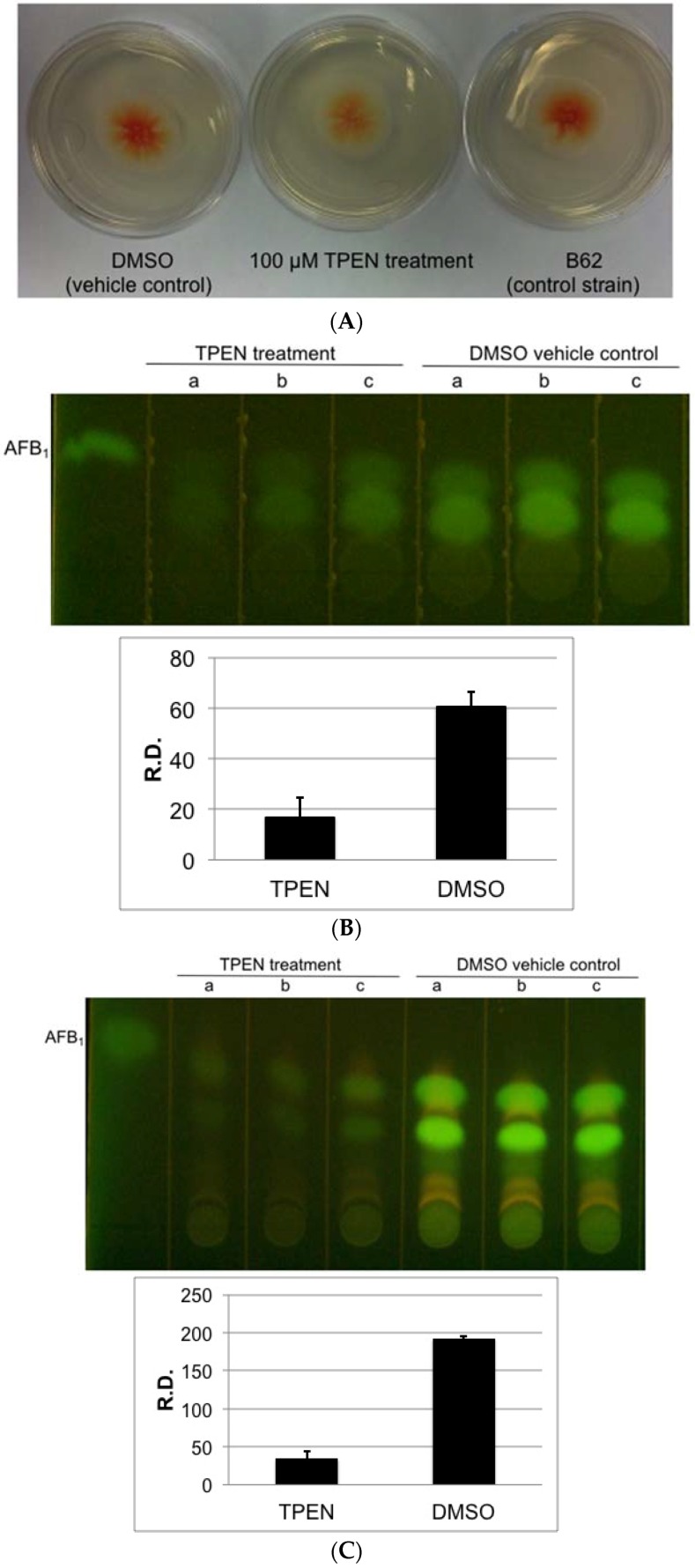
Zinc chelator, TPEN reduces toxin accumulation in *A. parasiticus* B62. (**A**) Visual accumulation of norsolorinic acid on solid PDA growth medium. *A. parasiticus* B62 was center inoculated onto PDA solid medium containing 100 μM TPEN or equal quantity of DMSO vehicle control, and incubated at 30 °C in the dark for 50 h; (**B**) TLC analysis (top panel) and densitometry analysis (bottom panel) of aflatoxin accumulation on solid PDA medium. Each lane represents an extract from one colony plus agar. A total of three colonies (triplicate samples) were grown in the presence of either 100 μM TPEN or DMSO (vehicle control); (**C**) TLC analysis (top panel) and densitometry analysis (bottom panel) of aflatoxin on solid GMS medium. Each lane represents an extract from one colony plus agar. A total of three colonies (triplicate samples) were grown in the presence of either 200 μM TPEN or DMSO (vehicle control). Error bars represent the standard error of the mean obtained from triplicate samples. Lower case letters a, b, c in the top panel indicate triplicate samples for each experimental condition. AFB_1_, aflatoxin B1. The fluorescence intensity was assessed using ImageJ 1.49v software (NIH, Bethesda, MD, USA). R.D., relative density as measured by ImageJ. Data are presented as the Mean ± SE, *N* = 3.

To simplify visualization of toxin accumulation, we utilized an *A. parasiticus* B62 mutant that accumulates the orange pigment norsolorinic acid as a reliable and rapid screening tool for assessment of the effect of TPEN on aflatoxin biosynthesis (Figure 1A) [25]. TPEN treatment reduced aflatoxin accumulation (as observed by densitometry conducted on TLC analysis) up to 3-fold compared to vehicle control on solid PDA medium (Figure 1B).

As proof of concept, we also investigated the effect of TPEN on aflatoxin production in *A. parasiticus* B62 on a chemically defined minimal medium, GMS. In preliminary experiments, we established a similar TPEN dose response on aflatoxin accumulation on GMS and found 200 μM to be an effective inhibitory dose for subsequent experiments on GMS. Similar to observations on solid PDA media, 200 μM TPEN significantly decreased aflatoxin B_1_ (AFB_1_) levels up to 6-fold compared to vehicle control on solid GMS media without affecting fungal growth (Figure 1C and Figure S3). The magnitude of TPEN effect on aflatoxin levels observed in GMS was far greater than on PDA media.

In contrast, preliminary dose response experiments with DMPS (range from 100 μM to 1000 μM) demonstrated little or no effect of norsolorinic acid reduction on both PDA and GMS (Figure S4). To confirm this observation, *A. parasiticus* wild-type SU-1 was grown for 5 days on solid PDA media with 300 μM DMPS or vehicle control (water). 300 μM DMPS did not affect aflatoxin accumulation on GMS as measured by TLC analysis (Figure S5).

### 2.2. Effect of TPEN on Aflatoxin Transcript Accumulation in *A. parasiticus* SU-1

**Figure 2:**
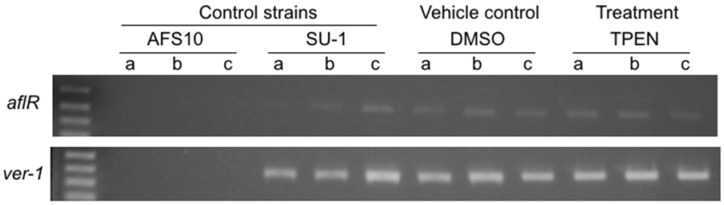

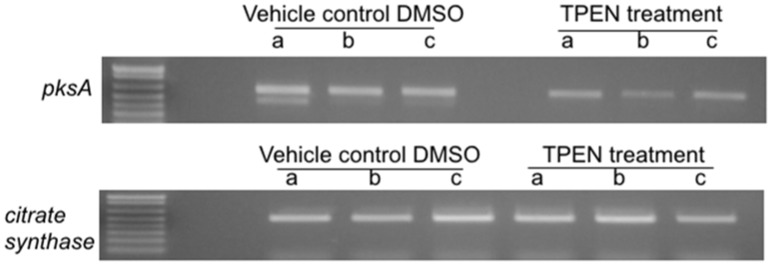
Effect of TPEN on aflatoxin transcript accumulation in *A. parasiticus* SU-1. *A. parasiticus* SU-1 conidiospores (10^4^ spores) were center inoculated onto solid PDA medium and plates were incubated at 30 °C in the dark for 50 h. Triplicate fungal colonies were grown either with 100 μM TPEN or with DMSO (vehicle control). Additional controls included AFS10 (*aflR* knockout) and SU-1 (wild type) grown under standard conditions (30 °C in the dark). Total RNA was extracted from frozen mycelium from three independent colonies (indicated by lowercase letters a, b, c) as described in Materials and Methods. Semi-quantitative RT-PCR performed on total RNA with gene specific PCR primers (see Methods, Table 1). PCR products were separated by electrophoresis on a 1% agarose gel. Citrate synthase, a constitutively expressed gene was used as a positive control.

To determine whether TPEN treatment affected aflatoxin accumulation at the level of gene expression, *A. parasiticus* SU-1 was grown under standard growth conditions on PDA with 100 μM TPEN or with DMSO (vehicle control), RNA extracted and cDNA prepared for transcript analysis by semi-qPCR. TPEN treatment did not alter transcript levels of *aflR* (one key positive pathway regulator and transcription factor) as well as levels of *ver-1* (encodes an aflatoxin enzyme) as detected by semi qPCR analysis (Figure 2) [26,27]. To eliminate the possibility that TPEN could be targeting and regulating aflatoxin gene transcripts prior to *nor-1* (*A. parasiticus* B62 mutant harbors a mutation in *nor-1* and accumulates NA), we analyzed levels of a polyketide synthase encoding gene, *pksA* [25]. We observed a modest 2-fold decrease in *pksA* transcript levels, which could at least in part explain the observed decreases in aflatoxin accumulation (Figure 2). However, due to the significant inhibition of aflatoxin and/or NA production by TPEN treatment, we hypothesized that the effect of TPEN was not completely at the level of gene expression.

### 2.3. TPEN Increases Intracellular Zinc Levels

**Figure 3:**
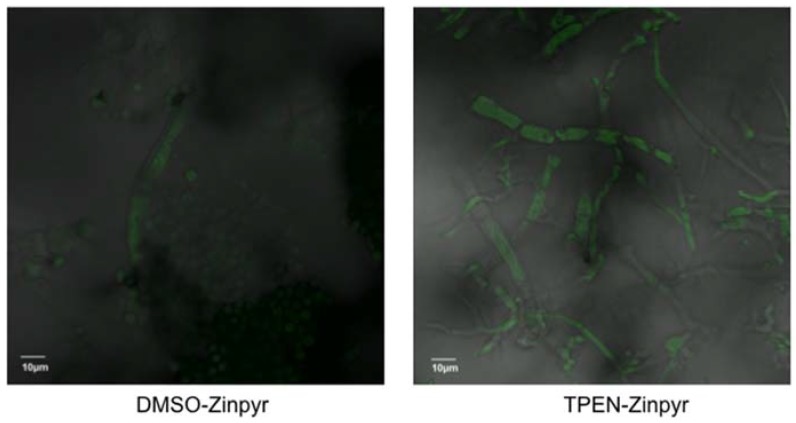
TPEN increases intracellular zinc levels. *A. parasiticus* wild-type SU-1 spores were center inoculated onto solid GMS media with and without 200 μM TPEN and were grown at 30 °C for 5 days. Plugs containing fungal colony and media were taken from GMS with and without TPEN and incubated with 10 μL Zinpyr-1 (5 μM) probe for 30 min at 37 °C and examined under a fluorescent microscope. The stained cells were observed under the fluorescent microscope as described in Methods. The accumulation of green fluorescent Zinpyr-1 probe was detected within the intracellular matrix of fungal mycelia. Bar 10 μm.

What are the mechanisms by which TPEN affects the down-regulation of aflatoxin accumulation independent of gene expression in *A. parasiticus*? We further hypothesized that TPEN is able to chelate extracellular (for example, 5 μM Zn^2+^ supplemented to solid GMS medium) and intracellular zinc to exhibit an effect on aflatoxin biosynthesis. To test this hypothesis, we used a cell permeable fluorescein-based Zn^2+^ sensor called Zinpyr-1 (ZP-1) to observe and track possible changes in intracellular zinc levels caused by TPEN treatment [28]. Interestingly, 200 μM TPEN increased overall fluorescence intensity of ZP-1 (increased in green fluorescence observed within fungal mycelia) compared to DMSO control as observed by confocal microscopy (Figure 3). These data suggested that TPEN binds intracellular zinc making it less available for biological function in the fungus.

### 2.4. TPEN Affects Toxin Accumulation in *A. parasiticus* Grown on Aflatoxin-Inducing Peanut-Derived Medium but Not in Sunflower Seed-Derived Medium

**Figure 4:**
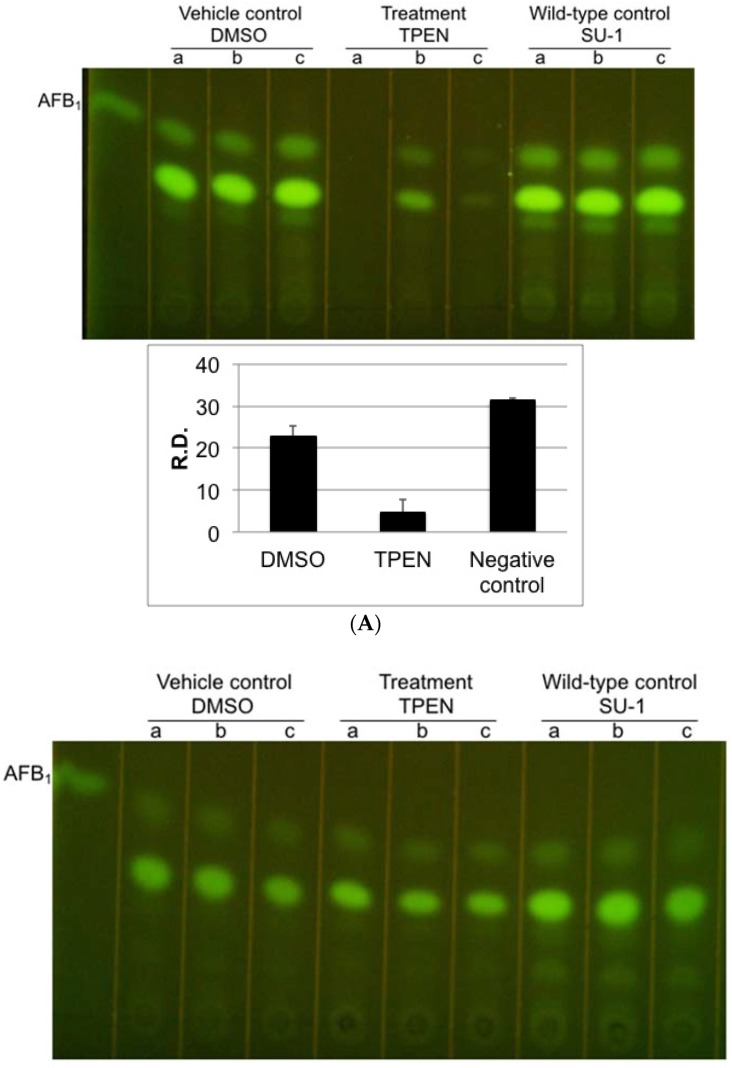

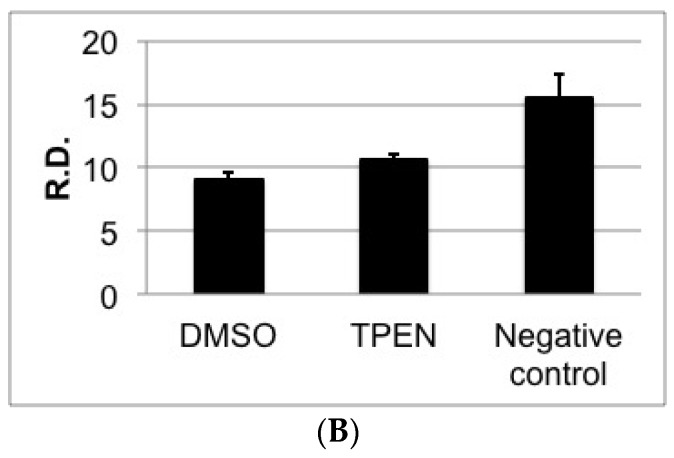
TPEN affects toxin accumulation in *A. parasiticus* grown on aflatoxin-inducing medium derived from natural substrates. (**A**) TLC analysis of aflatoxin accumulation on solid whole ground peanut medium. Each lane represents an extract from one colony plus agar. A total of three colonies (triplicate samples) were grown in the presence of either 200 μM TPEN or vehicle (DMSO); (**B**) TLC analysis of aflatoxin accumulation on solid whole ground sunflower seed medium. Each lane represents an extract from one colony plus agar. A total of three colonies (triplicate samples) were grown in the presence of either 200 μM TPEN or vehicle (DMSO) Wild-type SU-1 with no addition (negative control) was grown under the same conditions. Lower case letters a, b, c indicate triplicate samples for each experimental condition. AFB_1_: aflatoxin B_1_ standard (Sigma). The fluorescence intensity was assessed using ImageJ 1.49v software (NIH, Bethesda, MD, USA). R.D., relative density as measured by ImageJ. Data are presented as the Mean ± SE, *N* = 3 except TPEN treatment (Figure 4 bottom panel lane ‘a’, *N* = 2), ImageJ did not detect visible fluorescence.

In nature, peanuts and sunflower seeds provide favorable conditions for aflatoxin production both during growth and in storage [29,30]. To assess the possibility of using zinc chelators or zinc limitation to block aflatoxin production on food and feed crops, we tested the efficacy of TPEN on aflatoxin-inducing peanut and sunflower seed-derived media. Our work and others previously showed that peanuts are a more favorable source for aflatoxin production compared to other tree nuts (Figure S6) [31]. Two hundred microliters of TPEN decreased aflatoxin accumulation (5-fold) without significantly affecting growth in peanut medium but not in sunflower seeds-derived medium (Figure 4 and Figure S7).

## 3. Discussion

Several lines of evidence suggest that fungal zinc metabolism is closely associated with fungal virulence. Levels of intracellular zinc impact the ability of fungal cells to cause infection in humans, animals, and plants by directly regulating virulence determinants (proteins) or by regulating zinc-dependent transcription factors required for infection [32]. Fungi in the genus *Aspergillus* are known for secondary metabolite production (gliotoxin, *A. fumigatus*; aflatoxin, *A. flavus* and *A. parasiticus*; sterigmatocystin and penicillin, *A. nidulans*). Because zinc is tightly regulated in the cell, intracellular levels of zinc could control activation of fungal virulence-associated proteins that subsequently regulate ability to cause infection.

Our data support the hypothesis that zinc chelation down-regulates aflatoxin biosynthesis on rich and defined medium. Similar to other compounds that function as chelators (for example EDTA), most chelators can bind more than one divalent cations. The use of chelators depends on type of application and the strength of binding affinity of these chelators. Compared to other zinc chelators, TPEN has the strongest affinity for Zn^2+^ (*K*_a_ = 10^15.58^ M^−1^) [33]. On the other hand, DMPS was reported to be a heavy metal chelator and binds to copper with the highest affinity [34]. In a study conducted using TPEN and DMPS in three different lung cell lines, the authors reported that DMPS was most effective at 2.0 mM (2000 μM) at inhibiting expression and secretion of immune mediators compared to TPEN that was most effective at 25 μM [35]. At the protein level (eotaxin chemokine), 2.0 mM DMPS caused a 40% inhibition of eotaxin protein secretion whereas the lowest dose 0.5 mM DMPS had little to no effect. 45% inhibition of eotaxin protein secretion was achieved using 25 uM TPEN and even at the lowest dose, 1 μM TPEN still resulted in a 30% inhibition of protein secretion.

TPEN has a strong inhibitory effect on toxin synthesis in *A. parasiticus* grown on a rich medium (PDA) and a chemically defined growth medium (GMS). Interestingly, the magnitude of down-regulation is far greater on GMS compared to PDA. One possible explanation for this difference is the level of Zn^2+^ in PDA compared to GMS. PDA contains a higher concentration of zinc than that added to GMS. GMS contains a defined concentration of Zn^2+^ (5 μM) whereas PDA contains zinc primarily from potatoes (ranges from 0.8 mg to 1.2 mg per 100 g) (USDA ARS National Nutrient Database, available online at http://www.ars.usda.gov/). Early studies by Buchanan highlight differential regulation of various components in glucose catabolism (levels of enzyme, acetyl CoA, NADP/NADPH ratio) of aflatoxin synthesis by various carbohydrate sources incorporated into growth medium [36,37]. The magnitude of aflatoxin gene expression and accumulation is strongly influenced by nutrients and media composition [38]. Therefore, the differences observed in the effect of TPEN on PDA compared to GMS could be due to how aflatoxin synthesis is regulated on a rich complex medium compared to a chemically-defined medium. Another explanation could be the sequestration of TPEN by components of PDA similar to glucans binding antibiotics limiting access of antibiotics to cellular targets [39]. However, to our knowledge, no published data has been conducted on the transport of TPEN/DMPS across cell membranes or the sequestration of media components by TPEN/DMPS making the zinc chelators unavailable for activity.

In analysis of zinc in mammalian cell culture systems, concentrations of TPEN treatment ranged from 5 to 30 μM [40,41]. Because TPEN is reported to induce cellular apoptosis, we conducted a preliminary dose response study using 20 to 500 μM TPEN. In support of TPEN’s apoptotic effect, 500 μM TPEN completely inhibited fungal growth on solid PDA media (Figure S1). Zinc limitation was also shown to reduce fungal growth in 6 different pathogenic fungi including *C. albicans* [14]. Therefore, zinc depletion could have fungistatic properties in addition to impacts on mycotoxins.

The direct effects of zinc limitation on fungal growth and secondary metabolism are not well understood. To begin to understand the mechanistic basis for downregulation of aflatoxin accumulation by TPEN, we analyzed transcript levels of the aflatoxin pathway genes *aflR* (a zinc-dependent key pathway transcription factor) and *ver-1* (an aflatoxin structural gene). Our initial hypothesis was that TPEN would chelate intracellular zinc and compete with zinc-dependent proteins such as AflR for Zn^2+^ therefore decreasing expression and/or function of a group of zinc-dependent proteins. However, we did not observe significant changes in *aflR* or *ver-1* expression grown under conditions outlined above. To rule out the possibility that TPEN could affect expression of genes in the aflatoxin gene cluster prior to *nor-1* (we used B62 strain with mutation in *nor-1* for rapid screening of norsolorinic acid production) we also analyzed *pksA* transcript levels, which represents an early aflatoxin pathway gene upstream from *nor-1.* We detected an approximately 2-fold decrease in *pksA* transcript accumulation which may in part explain the 3 to 6 fold decrease in aflatoxin levels detected in PDA and GMS media. TPEN could act on *pksA* transcription independent of AflR and the genes downstream from NA through another zinc-dependent transcription factor that impacts *pksA* but not genes downstream from NA. Based on gene expression analysis, our initial findings argue that the effect of TPEN inhibition on aflatoxin accumulation could occur independent of aflatoxin gene expression. Future studies will focus on alternative mechanisms by which TPEN could downregulate aflatoxin accumulation. First, it is possible that zinc limitation impacts aflatoxin biosynthesis by limiting the quantity of acetyl CoA which serves as the major substrate for aflatoxin biosynthesis. For example, analysis of the expression and/or function of genes involved in acetyl CoA accumulation or metabolite analysis of acetyl CoA/NADPH levels would provide an alternative mechanism to explain TPEN’s inhibitory effect on aflatoxin. In addition to conducting a time course RNA Seq analysis of the impact of TPEN on expression of genes directly involved in aflatoxin synthesis, it is also likely that TPEN acts at the post-transcriptional level to localize aflatoxin proteins to endosomes, an important site for aflatoxin synthesis.

## 4. Conclusion and Future Perspectives

The inhibitory effect of TPEN on aflatoxin accumulation in *A. parasiticus* was observed in laboratory growth medium but also on peanut seed-derived medium providing impetus to continue to study the utility of zinc limitation on reduction of aflatoxin levels in important food and feed crops. One means to impact zinc levels at the site of fungal infection is to modulate expression or activity of zinc scavenging proteins encoded by the fungus and the host plant. If one could tilt the competition for free zinc to the host plant and away from the fungus using plantibodies, inhibitory peptides, or zinc binding peptides expressed by the host plant, one could theoretically impact aflatoxin contamination levels in the field. This could have important implications to reduction of aflatoxin in frequently contaminated food and feed crops. From an application perspective, the differential effects of TPEN observed on peanut and sunflower seed-derived medium may also provide impetus to further analyze the complexities of nutrient composition (fat content of peanuts, phytic acid in soybeans), lot-to-lot variation, and differences in food structural matrices.

## 5. Material and Methods

### 5.1. Fungal Strains and Growth Conditions

*Aspergillus parasiticus* strains SU-1 (ATCC 56775, a wild-type aflatoxin producer) and B62, a mutant strain-derived from SU-1 were used in the current study. *A. parasiticus* B62 carries a single amino acid substitution in *nor-1*, which results in accumulation of the brightly colored orange pathway intermediate norsolorinic acid (NA) and a small quantity of aflatoxin [25]. To simplify visualization of toxin accumulation, the accumulation of NA at the bottom of the fungal colony was used as a reliable and rapid screening tool for assessment of the effect of TPEN on aflatoxin biosynthesis. *A. parasiticus*, AFS10 (knockout of *aflR*, a key positive regulator of aflatoxin synthesis, produces no aflatoxin) was used as a control strain for gene expression analysis. AFS10 does not express *aflR*, *ver-1* or *pksA* at detectable levels.

Solid potato dextrose agar (PDA; BD Difco™, Sparks, MD, USA), a rich medium, and glucose minimal medium supplemented with sucrose and 5 μM Zn^2+^ glucose minimal salts (GMS), a chemically defined medium were used as aflatoxin-inducing growth media [36]. Two additional aflatoxin-inducing mimic media derived from whole ground peanuts and sunflower seeds were used to replicate growth conditions in the field and in storage. Whole raw peanuts or whole sunflower seeds were ground to a fine powder in a Ninja® blender. 10 g of this was added to 100 mL of water in a glass beaker which was then brought to a boil for 5 min over a Bunsen burner. The heated mixture was returned to the blender and blended for 1 min. The remaining solid pieces were removed using several layers of cheesecloth. The collected liquid was brought to a final volume of 250 mL, mixed with 3.75 g of agar, and autoclaved prior to pouring into petri dishes.

Conidiospores (10^4^ spores/plate) were center inoculated onto small Petri dishes (15 mm × 60 mm) containing 10 mL of each respective medium and grown under standard growth conditions at 30 °C in the dark for appropriate times (PDA, 50 h; GMS, peanut and sunflower seeds-derived media, 5 days). These time points were selected based on maximal production of aflatoxin and/or norsolorinic acid after fungal growth on various solid growth medium.

*N,N,N′,N′*-Tetrakis (2-pyridylmethyl) ethylenediamine (TPEN; Sigma-Aldrich, St. Louis, MO, USA) was dissolved in DMSO (Sigma-Aldrich, St. Louis, MO, USA) to create varying concentrations of TPEN in the same volume of DMSO for application onto solid media 50 μL of each TPEN concentration or DMSO vehicle control were pipetted onto the media surface and spread evenly using a bent glass rod to coat the entire surface. Plated concentrations of TPEN ranged from 20 μM to 500 μM. Plates were allowed to dry in the biosafety hood with lids slightly ajar for 10 min to allow the TPEN/DMSO to absorb into the media. Spores where then center inoculated and the plates incubated as indicated previously. Sodium 2,3-dimercaptopropanesulfate monohydrate (DMPS; Sigma-Aldrich, St. Louis, MO, USA) was dissolved in water to create varying concentrations of DMPS in the same volume of water for application onto solid media. 50 μL of each DMPS concentration or water vehicle control were pipetted onto the media surface and spread evenly using a bent glass rod to coat the entire surface. Plated concentrations of DMPS ranged from 100 μM to 500 μM. Plates were allowed to dry in the biosafety hood with lids slightly ajar for 10 min to allow the DMPS/water to absorb into the media. Spores where then center inoculated and the plates incubated as indicated previously.

Evaluation of fungal growth was estimated by colony diameter after 50 h (PDA treatment) or 5 days (GMS treatment) of incubation. Colony diameter was measured in two perpendicular directions, and the measurements for all colonies in each group were presented as mean ± standard error (SE) [23].

### 5.2. Aflatoxins B_1_, B_2_, G_1_, and G_2_ and Norsolorinic Acid Extraction, Detection, and Analysis

Aflatoxins B_1_, B_2_, G_1_, and G_2_ were extracted from one whole fungal colony together with the agar medium [4] and analyzed by TLC as previously described [42]. Aflatoxin B_1_ purchased from Sigma-Aldrich (St. Louis, MO, USA) was used as the standard for TLC analysis.

Each lane from each TLC run represents an extract from one colony plus agar. A total of three colonies (triplicate samples) were grown for each treatment condition. Error bars obtained from densitometry analysis represent the standard error of the mean obtained from triplicate samples. The fluorescence intensity was assessed using ImageJ 1.49v software (NIH, Bethesda, MD, USA) based on corrected total cell fluorescence (CTCF) = Integrated Density − (Area of selected cell × Mean fluorescence of background readings) to measure aflatoxin production on TLC [43,44].

### 5.3. Total RNA Extraction and Gene Expression Analysis

To allow rapid harvest and immediate freezing of whole colonies in liquid nitrogen for RNA extraction, conidiospores (10 μL containing 10^4^ spores) of *A. parasiticus* SU-1 were center inoculated onto sterile cellophane disks placed on solid GMS medium containing 100 μM TPEN or DMSO and grown 50 h at 30 °C in the dark. Individual whole fungal colonies were ground in liquid nitrogen using a mortar and pestle [4] and total RNA isolated via the TRIzol method (Invitrogen, Carlsbad, CA, USA) according to manufacturer’s instructions. The extracted RNA was treated with RNase-free DNase (Qiagen, Valencia, CA, USA) and the quality determined with an Agilent 2100 Bioanalyzer (Agilent Technologies, Santa Clara, CA, USA). For semi-qPCR analyses, total RNA (1 μg) was treated with gDNA WipeOut and cDNA prepared with the QuantiTect reverse transcription kit (Qiagen, Valencia, CA, USA) according to the manufacturer’s instructions.

Subsequent PCR was conducted using 2 μL of cDNA as the template using the following thermocycler (Perkin Elmer GeneAmp PCR system 2400, Perkin Elmer, Waltham, MA, USA) parameters: Initial denaturation at 94 °C for 5 min; 30 cycles at 94 °C for 1 min, 60 °C for 2 min, and 72 °C for 1 min; and final elongation at 72 °C for 10 min. Table 1 shows the primer pairs used for *aflR*, *ver-1*, *pksA*, and citrate synthase transcript analysis. PCR products were separated by electrophoresis on a 1% agarose gel. Band intensities for aflatoxin transcripts were compared to the level of citrate synthase intensity, a housekeeping gene under the same conditions. Citrate synthase transcript accumulation as detected by semi-qPCR analyses did not change under growth conditions previously published [19] or in the current study.

### 5.4. Zinpyr-1 and Confocal Scanning Laser Microscopy

Zinpyr-1 (Santa Cruz Biotech, Santa Cruz, CA, USA), a zinc selective, cell-permeable, fluorescent probe was used to observe intracellular zinc levels. Zinpyr-1 was dissolved in DMSO to create a stock solution to be applied at a 5 μM working concentration. GMS medium was prepared, treated and incubated with either 200 μM TPEN or DMSO as described above. *A. parasiticus* wild-type SU-1 spores (10^4^ spores) were center inoculated onto GMS containing TPEN or DMSO and incubated at 30 °C for 5 days. After 5 days, one round plug including both media and fungal growth, of a consistent size, were taken from each colony just inside the outer edge of the growth. Under minimal light exposure, the plugs were placed into a two-chambered cover glass microscope slide (Thermo Scientific Lab-Tek II, Waltham, MA, USA) and 10 μL of the probe or DMSO control was pipetted immediately on to the plug. The lids were placed on the chambers and the slides were incubated in a 37 °C incubator for 30 min to allow the probe to absorb and adhere to the zinc. Each slide was then placed onto a laser scanning confocal microscope (Olympus FluoView™ FV1000, Olympus America Inc., Center Valley, PA, USA) and images captured.

## Figures and Tables

**Table 1:** Primer sequences used for transcript analysis.

Gene	Primer Sequence	Reference
*aflR*	F-5′ TGAGAACGATAAGGACGAC 3′	[4]
	R-5′ CATCCTCAATCGAATCAAC 3′	
*ver-1*	F-5′ TTGTATCGTTCGGTCACC 3′	[45]
	R-3′ GGTTCAAAGGAGAGAGCC 3′	
*citrate synthase*	F-5′ TGCAGTCCGTTGCCTTCAATG 3′	[5]
	R-5′ TAGCGTAGGCCTTGGCGAAAG 3′	
*pksA*	F-5′ GTTCCTTGGCCGCTGTG 3′	[45]
	R-5′ AAAGGCGTGGCAGTCC 3′	

## Supplementary Materials

The following are available online at www.mdpi.com/2072-6651/8/6/171/s1., Figure S1: Five hundred micromolar (μM) TPEN causes complete fungal growth inhibition. Visual observation of fungal growth on solid PDA growth medium. *A. parasiticus* B62 was center inoculated onto PDA solid medium containing 500 μM TPEN, 100 μM TPEN, or equal quantity of DMSO vehicle control, and incubated at 30 °C in the dark for 50 h. Lower case letters a, b, c in the top panel indicate triplicate samples for each experimental condition. (Yellow) 500 μM TPEN; (Red) 100 μM TPEN; (Blue) DMSO (vehicle); Figure S2: TPEN treatment does not affect *A. parasiticus* growth on PDA. Conidiospores (1 × 10^4^ spores/plate) of *A. parasiticus* B62 were center inoculated onto PDA and grown for 50 h at 30 °C in the dark. (**A**) Evaluation of growth was performed by colony diameter as described in “Materials and Methods”. The fungus was exposed to following conditions: B62 only control, DMSO vehicle control, 20 μM and 100 μM TPEN treatment. Three independent colonies were analyzed for each treatment condition. Data are presented as mean ± SE; Dose response effect of TPEN on toxin accumulation in *A. parasiticus* wild-type SU-1. (**B**) TLC analysis of aflatoxin accumulation on solid PDA medium. *A. parasiticus* SU-1 was center inoculated onto PDA solid medium containing varying concentrations of TPEN or equal quantity of DMSO vehicle control, and incubated at 30 °C in the dark for 50 h. Each lane represents an extract from one colony plus agar. Lower case letters a, b, c in the top panel indicate triplicate samples (total of three colonies) for each experimental condition. AFB_1_: aflatoxin B_1_ standard (Sigma); Figure S3: Two hundred micromolar (μM) TPEN treatment does not affect *A. parasiticus* growth on GMS. Conidiospores (1 × 10^4^ spores) of *A. parasiticus* B62 were center inoculated onto GMS and grown for 5 days at 30 °C in the dark. Evaluation of growth was performed by colony diameter as described in “Materials and Methods”. The fungus was exposed to following conditions: B62 only control, DMSO vehicle control, 100 μM, 200 μM, 300 μM, and 400 μM TPEN treatment. Three independent colonies were analyzed for each treatment condition. Data are presented as mean ± SE; Figure S4**:** Dose response effect of DMPS on toxin accumulation in *A. parasiticus* B62 strain. (**A**) Visual accumulation of norsolorinic acid on solid PDA growth medium. *A. parasiticus* B62 (1 × 10^4^ spores) was center inoculated onto PDA solid medium containing 100 μM and 200 μM DMPS or equal quantity of water vehicle control, and incubated at 30 °C the dark for 50 h; (**B**) visual accumulation of norsolorinic acid on solid PDA growth medium. *A. parasiticus* B62 (1 × 10^4^ spores) was center inoculated onto PDA solid medium containing 300 μM to 1000 μM DMPS or equal quantity of water vehicle control, and incubated at 30 °C the dark for 50 h; (**C**) visual accumulation of norsolorinic acid on solid GMS growth medium. *A. parasiticus* B62 (1 × 10^4^ spores) was center inoculated onto GMS solid medium containing 100 μM and 200 μM DMPS or equal quantity of water vehicle control, and incubated at 30 °C the dark for 5 days; (**D**) visual accumulation of norsolorinic acid on solid GMS growth medium. *A. parasiticus* B62 (1 × 10^4^ spores) was center inoculated onto GMS solid medium containing 300 μM to 500 μM DMPS or equal quantity of water vehicle control, and incubated at 30 °C the dark for 5 days. Lower case letters a, b, c in the top panel indicate triplicate samples (total of three colonies) for each experimental condition. *A. parasiticus* B62 strain was used as a negative control; Figure S5: Effect of 300 μM DMPS on toxin accumulation in *A. parasiticus* wild-type SU-1. TLC analysis of aflatoxin accumulation on solid GMS medium. *A. parasiticus* SU-1 was center inoculated onto GMS solid medium containing 300 μM of DMPS or equal volume of vehicle control (water), and incubated at 30 °C in the dark for 5 days. Each lane represents an extract from one colony plus agar. Lower case letters a, b, c in the top panel indicate triplicate samples (total of three colonies) for each experimental condition. AFB_1_: aflatoxin B_1_ standard (Sigma); Figure S6**:** Peanut is a preferred source for aflatoxin production compared to sunflower media. TLC analysis of aflatoxin accumulation on solid whole ground peanut medium and sunflower seeds-derived medium. Each lane represents an extract from one colony plus agar. Lower case letters a, b, c indicate triplicate samples for each experimental condition. AFB_1_: aflatoxin B_1_; Figure S7**:** Two hundred micromolar **(**μM) TPEN does not significantly affect growth in *A. parasiticus* SU-1 strain. Visual observation of fungal growth on solid peanut-derived growth medium (see Methods). *A. parasiticus* SU-1 was center inoculated onto peanut medium containing 200 μM TPEN or equal quantity of DMSO vehicle control, and incubated at 30 °C in the dark for 5 days. Lower case letters a, b, c in the left panel indicate triplicate samples for each experimental condition.
