# Supplementary material for: Effects of Zinc Chelators on Aflatoxin Production in Aspergillus parasiticus
Source: Toxins (Basel). 2016 Jun 2;8(6):171. doi: 10.3390/toxins8060171 (PMC4926138; doi:10.3390/toxins8060171)
Supplement: Supplementary file 1 [file toxins-08-00171-s001.pdf]

Supplementary Materials: Effects of Zinc Chelators on Aflatoxin Production in *Aspergillus parasiticus*

Josephine Wee, Devin M. Day and John E. Linz

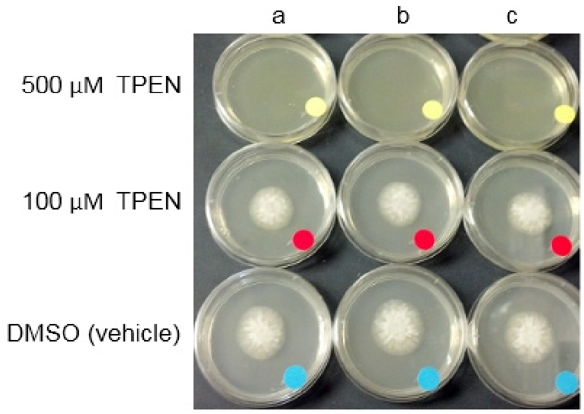

Figure S1. Five hundred micromolar (uM) TPEN causes complete fungal growth inhibition. Visual observation of fungal growth on solid PDA growth medium. *A. parasiticus* B62 was center inoculated onto PDA solid medium containing 500 μM TPEN, 100 μM TPEN, or equal quantity of DMSO vehicle control, and incubated at 30 °C in the dark for 50 h. Lower case letters a, b, c in the top panel indicate triplicate samples for each experimental condition. (Yellow) 500 μM TPEN; (Red) 100 μM TPEN; (Blue) DMSO (vehicle).

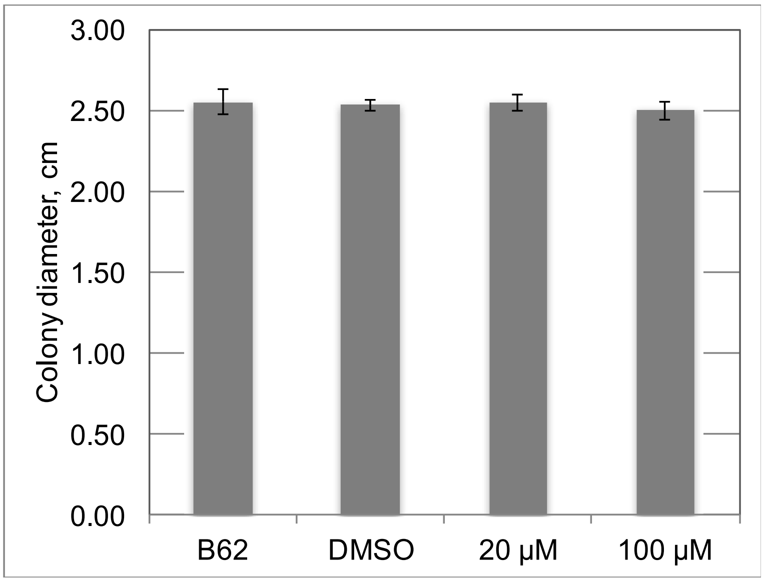

(**A**)

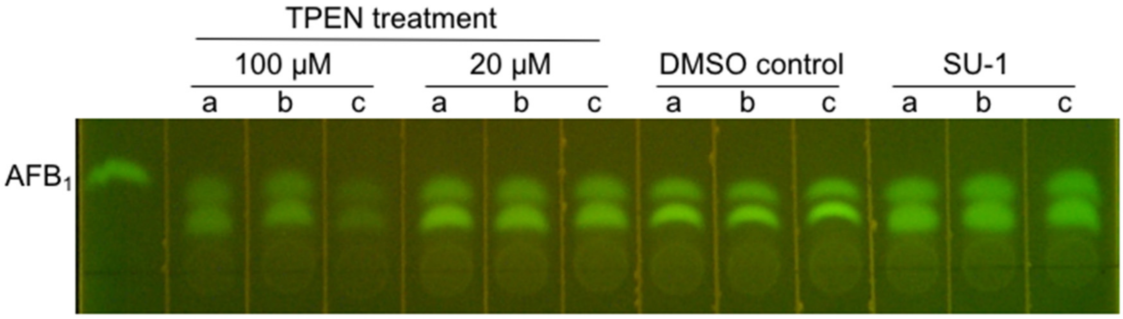

(**B**)

Figure S2. TPEN treatment does not affect *A. parasiticus* growth on PDA. Conidiospores (1×10^4 spores/plate) of *A. parasiticus* B62 were center inoculated onto PDA and grown for 50 h at 30 °C in the dark. (**A**) Evaluation of growth was performed by colony diameter as described in "Materials and Methods". The fungus was exposed to following conditions: B62 only control, DMSO vehicle control, 20 μM and 100 μM TPEN treatment. Three independent colonies were analyzed for each treatment condition. Data are presented as mean ± SE; Dose response effect of TPEN on toxin accumulation in *A. parasiticus* wild-type SU-1. (**B**) TLC analysis of aflatoxin accumulation on solid PDA medium. *A. parasiticus* SU-1 was center inoculated onto PDA solid medium containing varying concentrations of TPEN or equal quantity of DMSO vehicle control, and incubated at 30 °C in the dark for 50 h. Each lane represents an extract from one colony plus agar. Lower case letters a, b, c in the top panel indicate triplicate samples (total of three colonies) for each experimental condition. AFB_1: aflatoxin B_1 standard (Sigma).

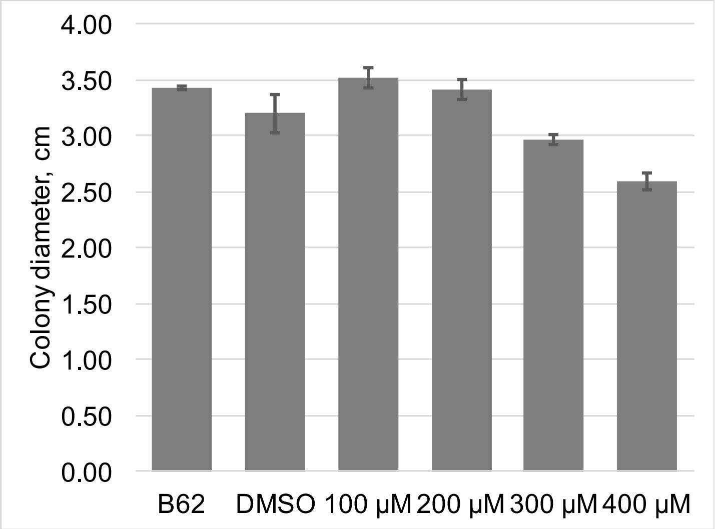

Figure S3. Two hundred micromolar (uM) TPEN treatment does not affect *A. parasiticus* growth on GMS. Conidiospores (1 × 10^4 spores) of *A. parasiticus* B62 were center inoculated onto GMS and grown for 5 days at 30 °C in the dark. Evaluation of growth was performed by colony diameter as described in "Materials and Methods". The fungus was exposed to following conditions: B62 only control, DMSO vehicle control, 100 µM, 200 µM, 300 µM, and 400 µM TPEN treatment. Three independent colonies were analyzed for each treatment condition. Data are presented as mean ± SE.

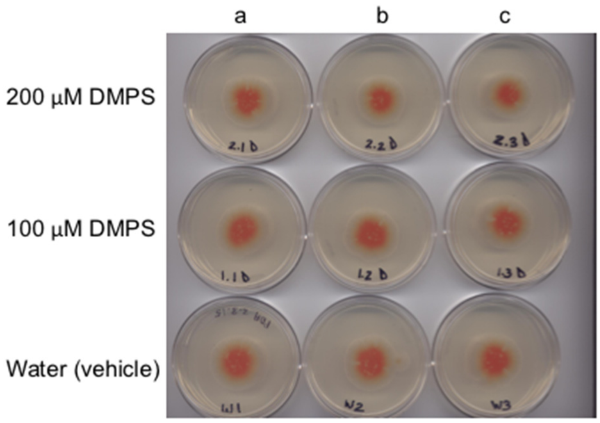

(**A**)

Figure S4. *Cont.*

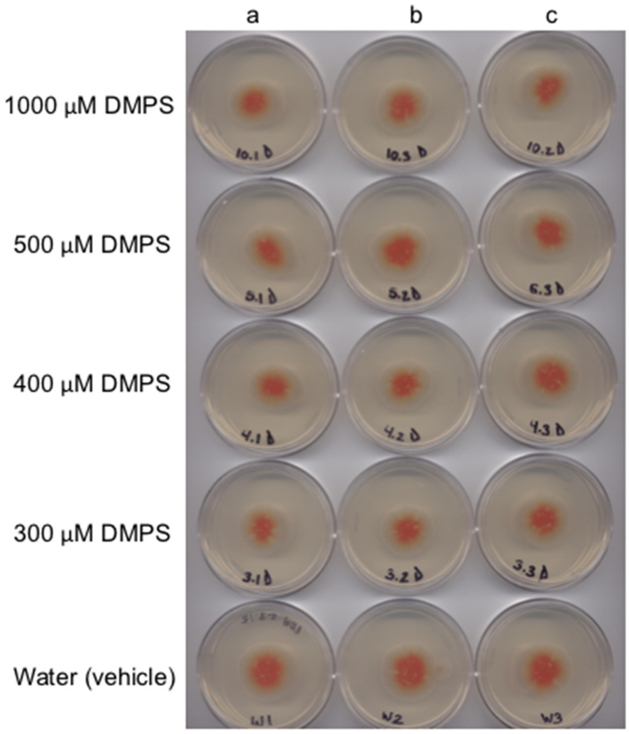

(**B**)

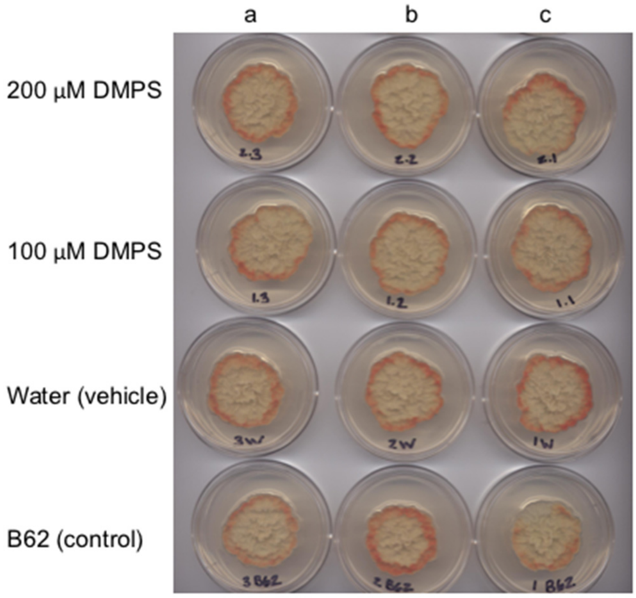

(**C**)

Figure S4. *Cont.*

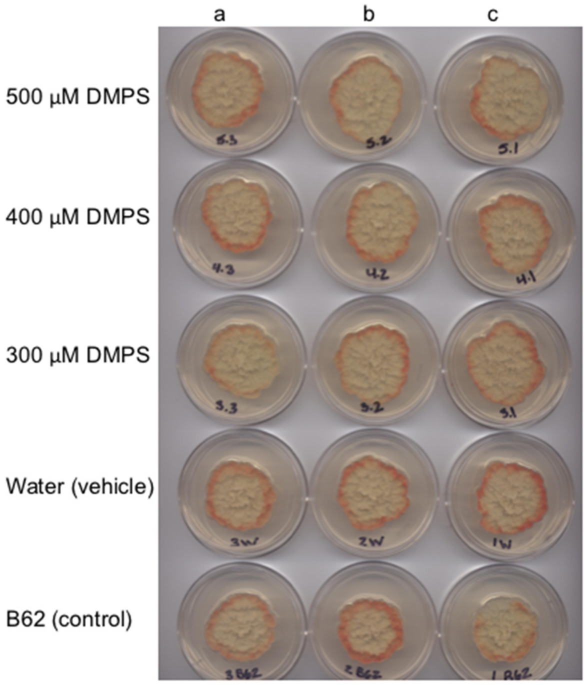

(**D**)

Figure S4. Dose response effect of DMPS on toxin accumulation in *A. parasiticus* B62 strain. (**A**) Visual accumulation of norsolorinic acid on solid PDA growth medium. *A. parasiticus* B62 (1×10^4 spores) was center inoculated onto PDA solid medium containing 100 μM and 200 μM DMPS or equal quantity of water vehicle control, and incubated at 30 °C the dark for 50 h; (**B**) visual accumulation of norsolorinic acid on solid PDA growth medium. *A. parasiticus* B62 (1×10^4 spores) was center inoculated onto PDA solid medium containing 300 μM to 1000 μM DMPS or equal quantity of water vehicle control, and incubated at 30 °C the dark for 50 h; (**C**) visual accumulation of norsolorinic acid on solid GMS growth medium. *A. parasiticus* B62 (1×10^4 spores) was center inoculated onto GMS solid medium containing 100 μM and 200 μM DMPS or equal quantity of water vehicle control, and incubated at 30 °C the dark for 5 days; (**D**) visual accumulation of norsolorinic acid on solid GMS growth medium. *A. parasiticus* B62 (1×10^4 spores) was center inoculated onto GMS solid medium containing 300 μM to 500 μM DMPS or equal quantity of water vehicle control, and incubated at 30 °C the dark for 5 days. Lower case letters a, b, c in the top panel indicate triplicate samples (total of three colonies) for each experimental condition. *A. parasiticus* B62 strain was used as a negative control.

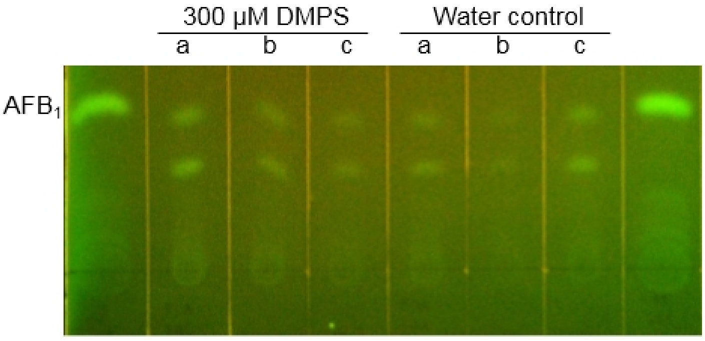

Figure S5. Effect of 300 μM DMPS on toxin accumulation in *A. parasiticus* wild-type SU-1. TLC analysis of aflatoxin accumulation on solid GMS medium. *A. parasiticus* SU-1 was center inoculated onto GMS solid medium containing 300 μM of DMPS or equal volume of vehicle control (water), and incubated at 30 °C in the dark for 5 days. Each lane represents an extract from one colony plus agar. Lower case letters a, b, c in the top panel indicate triplicate samples (total of three colonies) for each experimental condition. AFB_1: aflatoxin B_1 standard (Sigma-Aldrich, St. Louis, MO, USA).

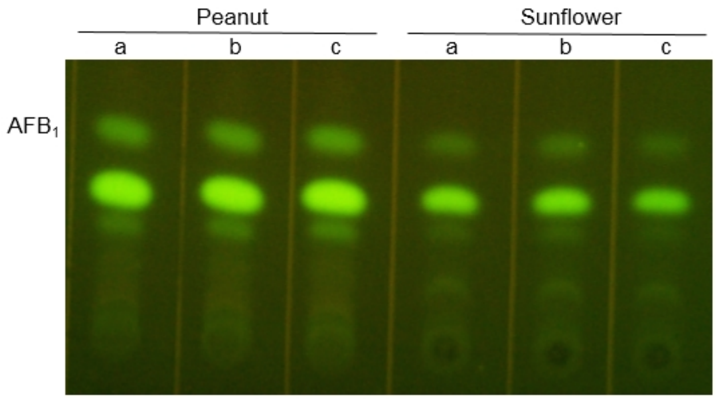

Figure S6. Peanut is a preferred source for aflatoxin production compared to sunflower media. TLC analysis of aflatoxin accumulation on solid whole ground peanut medium and sunflower seeds-derived medium. Each lane represents an extract from one colony plus agar. Lower case letters a, b, c indicate triplicate samples for each experimental condition. AFB_1: aflatoxin B_1.

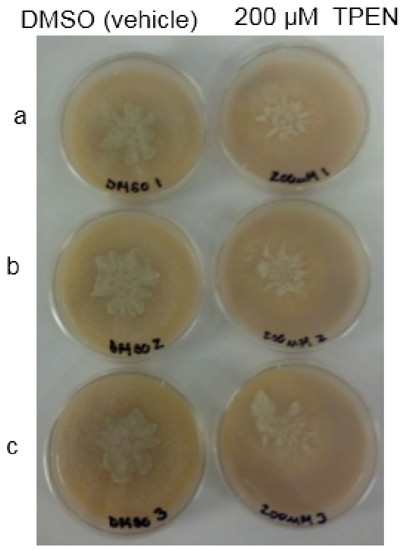

Figure S7. Two hundred micromolar (μM) TPEN does not significantly affect growth in *A. parasiticus* SU-1 strain. Visual observation of fungal growth on solid peanut-derived growth medium (see Methods). *A. parasiticus* SU-1 was center inoculated onto peanut medium containing 200 μM TPEN or equal quantity of DMSO vehicle control, and incubated at 30 °C in the dark for 5 days. Lower case letters a, b, c in the left panel indicate triplicate samples for each experimental condition.
